# Evolution: are the monkeys' typewriters rigged?

**DOI:** 10.1098/rsos.140172

**Published:** 2014-10-01

**Authors:** Michael R. Garvin, Anthony J. Gharrett

**Affiliations:** Fisheries Division, School of Fisheries and Ocean Sciences, University of Alaska Fairbanks, 17101 Point Lena Loop Road, Juneau, AK 99801, USA

**Keywords:** evolution, microsatellite, codon, mutation, molecular genetics

## Abstract

Evolution is presumed to proceed by random mutations, which increase an individual’s fitness. Increased fitness produces a higher survival rate for those individuals within populations and drives the variants to fixation over large timescales to produce new species. We recently identified positively selected sites in mitochondrial complex I in numerous, diverse taxa. In one taxon, a simple sequence repeat (SSR) encompassed the positively selected sites. We hypothesized a model in which: (i) slip-strand mis-pairing during replication due to the SSR increases the mutation rate at these sites, and (ii) a functional constraint at the protein level maintains the SSR and therefore a higher mutation rate at this site over large time scales to drive evolution. We tested this model by identifying SSRs in a mitochondrial-encoded protein in species from our previous work and determined that nearly all of the positively selected sites encompass an SSR. Furthermore, we show that our proposed model accounts for most of the mutations at neutral sites but it is probably the predominant mechanism at positively selected sites. This suggests that evolution does not proceed by simple random processes but is guided by physical properties of the DNA itself and functional constraint of the proteins encoded by the DNA.

## 2. Introduction

Darwinian evolution proceeds by way of mutation in the DNA of an individual and a selective force that acts on a physical trait that is based on the mutation, which renders the individual that carries it more fit than others in the population. This force increases the frequency of the mutation in a population until it becomes fixed, and that variant and others that are linked to it probably contribute to the formation of new species. A frequently repeated statement is that, ‘given an infinite amount of time and an infinite number of typewriters, an infinite number of monkeys could eventually type one of the works of Shakespeare’ and therefore by analogy, simple random mutation produces enough DNA variation on an evolutionary timescale for natural selection to act to produce new species. Much effort is currently directed at identifying genetic variants that underlie local adaptation and how those variants interact with the environment and selective forces to produce new species.

For example, in a recent analysis, we identified more than 80 candidate sites for positive selection in the coding sequences of the mitochondrial genomes from 237 diverse taxa [1]. Positive selection at a site was defined by two criteria: (i) a higher rate of non-synonymous substitutions compared with synonymous substitutions (*ω*) estimated with the software package PAML [2], and (ii) a significant change in the physico-chemical property of the amino acid substitutions at a site, which was determined with the program TreeSAAP [3].

The majority of the positively selected sites were located in a ‘helix HL connecting arm’ structure of the ND5 subunit that is part of mitochondrial complex I. This connecting arm is probably part of the biomechanical apparatus that coordinates the movement of protons across the inner mitochondrial membrane to generate ATP in metazoans [4]. We identified a likely functional role for the mutations at the molecular level and an environmentally relevant trait that may be a target of natural selection for these sites [1]. Therefore, it appeared that variation in a few functionally important amino acid sites occurred within the broader context of purifying selection and functional constraint in a highly conserved protein complex [5,6].

**Figure 1.**
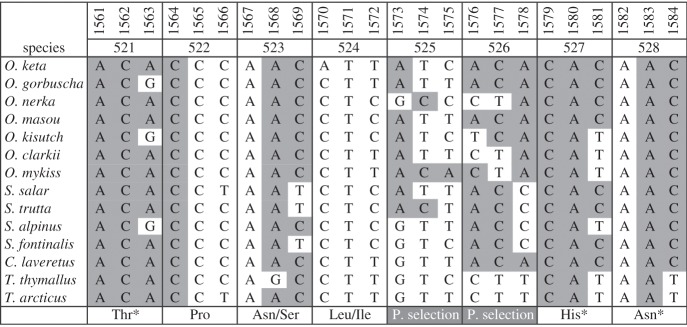
The (AC)_*n*_ di-nucleotide SSR (grey background with black letters) among species of Pacific salmon. The positively selected sites are indicated at the bottom with white letters on a grey background. Highly conserved amino acids that maintain the SSR are marked with an asterisk. Numbering in the first row is for the nucleotide number of the salmon ND5 gene and the second row is for the amino acid number. Accession numbers from top to bottom: AP010773, NC_010959, NC_008615, NC_008747, NC_009263, NC_006897, DQ288268, NC_001960, NC_010007, NC_000861, NC_000860, NC_002646, FJ853655, FJ872559.

Our original discovery of positive selection in mitochondrial DNA focused on Pacific salmon [7], which revealed some interesting properties in the DNA surrounding the selected site: (i) the selected site encompassed an (AC)_*n*_ di-nucleotide, simple sequence repeat (SSR); (ii) the selected site was adjacent to at least one highly conserved amino acid; (iii) that highly conserved amino acid was translated by codons that maintained at least one of the SSRs; and (iv) the (AC)_*n*_ SSRs appeared to be both gained and lost over the course of the evolution of this taxonomic group (figure 1).

**Figure 2.**
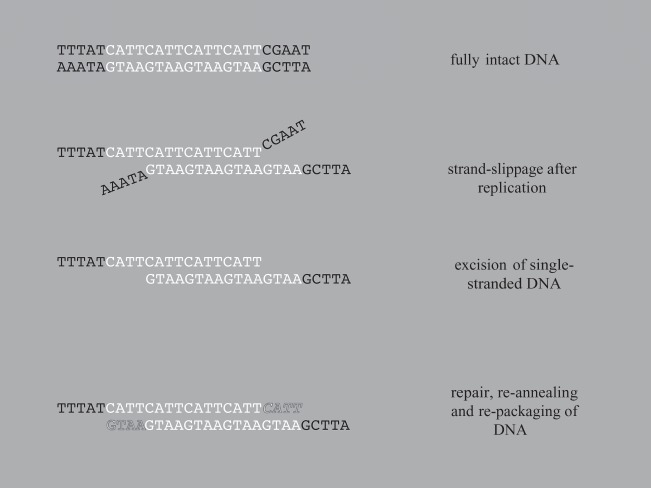
Graphical representation of slip-strand mis-pairing and repair that generates new repeat units in SSRs.

SSRs are segments of genomic DNA that are defined by a repeated nucleotide motif, the number of which is probably increased or decreased by slippage of the DNA strand during replication (figure 2) [8]. These SSRs are typically used for population genetic studies because they demonstrate many useful properties [9], which include: (i) the mechanism of contraction and expansion provides multiple alleles at a locus; (ii) multiple alleles can be generated over short timescales; and (iii) they occur primarily in non-coding regions of DNA and are often considered neutral [10], which is an important assumption for many evolutionary analyses. The last point, however, does not always hold [11]; SSRs can exist in regulatory regions of genes and the number of repetitive sequences may alter gene expression [9,11,12]. Introduction of new repeat units in coding DNA can also disrupt the function of genes but may also create new alleles and new protein variants.

The mismatch and base-excision repair activities that correct strand-slippage at SSRs operate in both mitochondrial and nuclear DNA [13–15] and may induce point mutations at those sites either owing to errors of the repair machinery or deamination of single-stranded DNA as a result of a highly oxidative environment that causes the transitions C <−>T and G <−> A [16,17]. In fact, SSRs have been shown to have higher substitution rates adjacent to the repeat unit [18–20], and point mutations within the repeat itself may be a mechanism to counteract SSRs in coding regions [21].

We hypothesize here that SSRs and protein functional constraint (for simplicity we will refer to these combined processes as ‘SRaF’) are the mechanism by which positively selected sites in mitochondrial DNA from diverse taxa are generated. SRaF is a result of two opposing forces: (i) slip-strand mis-pairing that occurs because of the SSR in the DNA sequence, which is followed by DNA repair that induces mutations; and (ii) constraint at the protein functional and structural levels that maintain at least one of the units of the SSR owing to high purifying selection [5,6]. These two properties ensure that the mutational process is maintained across evolutionary timescales because the SSR is preserved to produce a correctly functional protein. Our previously reported meta-analysis provides a set of data to test this hypothesis.

## 3. Material and methods

### 3.1 Sequence data

**Figure 3.**
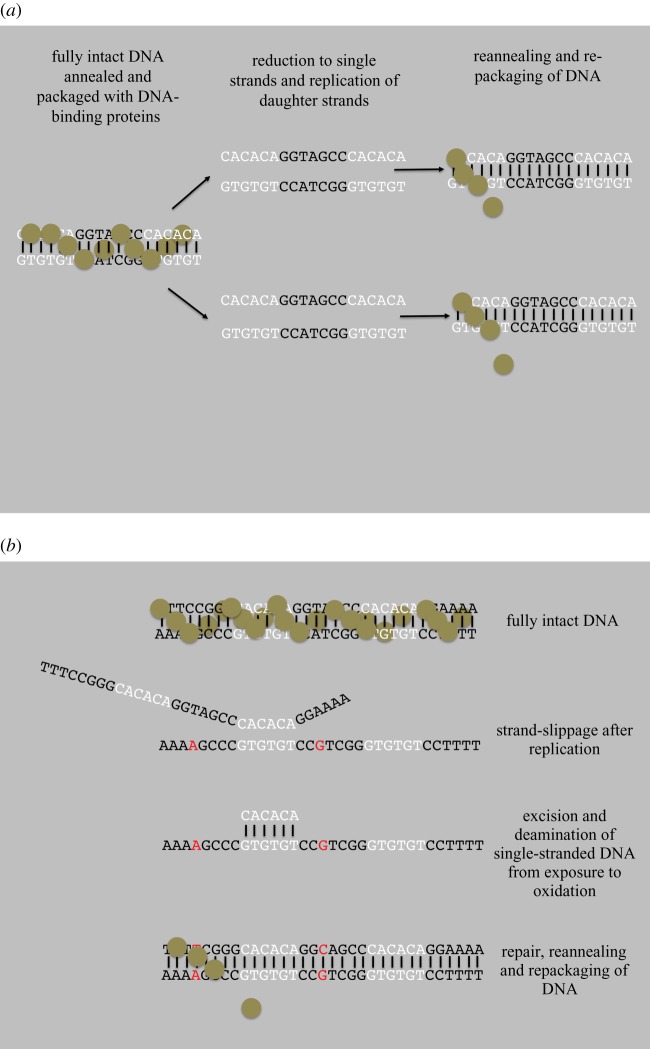
(*a*) Normal replication of mitochondrial DNA involves removal of DNA-binding proteins, generation of daughter strands and repackaging of DNA with protective DNA-binding proteins. (*b*) A representation of slip-strand mis-pairing that causes single-stranded DNA to be exposed long enough to produce deamination of nucleotides. Slippage is followed by repair of the daughter strand and repackaging of the DNA.

We searched each codon in the mitochondrial sequence data of the ND5 gene from 14 taxa that displayed positive selection from our meta-analysis [1] for the presence of an SSR that was: (i) part of the codon for that site, (ii) also observed in one or more of the adjacent codons in which (iii) the amino acid or acids were highly conserved across taxa. Because mitochondrial DNA is A–T rich, we only counted a di-nucleotide repeat as an SSR if at least two of the repeats were directly adjacent to each other. We only included polyN tracts as an SSR if there were at least four consecutive nucleotides. We counted mutations that occurred in the sequence between two SSRs as being in the ‘SSR’ category because they would also be implicated in the excision-repair process (see figure 3*a*,*b*). However, we did not count mutations that occurred in codons adjacent to a SSR that was not in between that one and another SSR because we could not be sure whether it was part of the excision-repair process. This produced a conservative estimate of the number of SSRs that are responsible for mutational events under our hypothesis.

### 3.2 Statistical test

The statistical significance between the categories of non-synonymous, synonymous and positively selected SSR sites was determined with a two-sample *t*-test in Microsoft Excel.

## 4. Results

**Figure 4.**
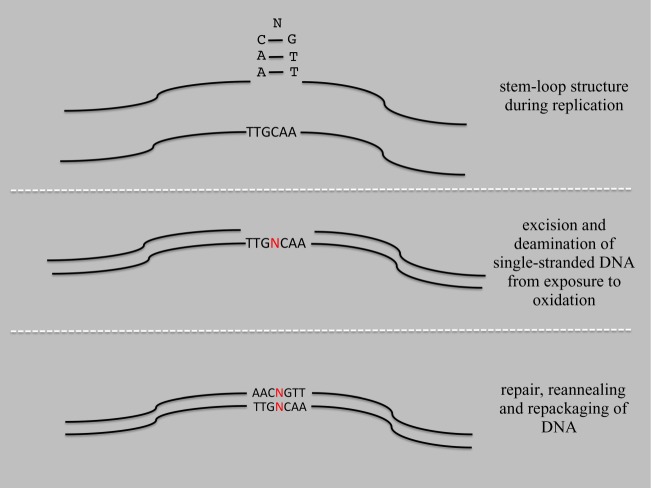
Representation of a possible mechanism of mutation generation from a stem-loop structure and the excision-repair mechanism that is also the basis of the SRaF model.

Our meta-analysis of positive selection in the mitochondrial genomes from diverse taxa [1] revealed 35 sites from 14 taxonomic groups that demonstrated positive selection in the biomechanical apparatus of the ND5 protein that was proposed to translocate protons across the inner mitochondrial membrane. We found evidence of an SSR in 34 of the 35 cases under our SRaF model (table 1;). The SSRs varied for the type of nucleotide, the number of repeats and complexity of the repeat. Because of the last property, we will now refer to the repeat units as ‘sequence repeats’. In the single case in which a sequence repeat was not present, the positively selected site was spanned by a sequence that could form a complementary loop structure (figure 4), which would also require excision and repair and may also initiate point mutations.

**Table 1.** Mutational mechanism for each of the 35 positively selected sites in our analysis of 237 coding mitogenome sequences.

species	mechanism	repeat unit	species	mechanism	repeat unit
*Anguilla*	SSR	(GTTA)_*n*_	Hominidae	SSR	(AAT)_*n*_
Argentinoidei	SSR	(ATT)_*n*_	Hominidae	SSR	poly C
Balaenopteridae	SSR	(CAAAT)_*n*_	*Hypsiglena*	SSR	(AACC)_*n*_
Balaenopteridae	SSR	(ATT)_*n*_	*Oryzias latipes*	SSR	(AAACC)_*n*_
Caprinae	SSR	(CT)_*n*_	*Oryzias latipes*	SSR	(AAACC)_*n*_
Caprinae	SSR	(ACCAATT)_*n*_	*Oryzias latipes*	SSR	(CAC)_*n*_
Caprinae	SSR	(ACC)_*n*_	*Oryzias latipes*	SSR	(TCAAT)_*n*_
Caprinae	complementary loop	ATCNNNNTAG	*Oryzias latipes*	SSR	poly C
Caprinae	SSR	(AATT)_*n*_	Otariidae	SSR	(TCAA)_*n*_
Caprinae	SSR	(CAA)_*n*_	Otariidae	SSR	(TCAA)_*n*_
Delphinidae	SSR	(CCT)_*n*_	Otariidae	SSR	(AAT)_*n*_
Delphinidae	SSR	poly C	Phocidae	SSR	(TAC)_*n*_
Delphinidae	SSR	(TCTAA)_*n*_	Phocidae	SSR	poly A
*Grus*	SSR	(GGCT)_*n*_	Phocidae	SSR	poly A
*Grus*	SSR	(CCT)_*n*_	Salmondiae	SSR	(AC)_*n*_
*Grus*	SSR	poly A	*Takifugu*	SSR	(CCAAC)_*n*_
*Grus*	SSR	(AAC)_*n*_	Ursidae	SSR	poly A & (TC)_*n*_
			Ursidae	SSR	(CTAA)_*n*_

These data support our hypothesis that slip-strand mis-pairing and functional constraints at the structural level of the protein are responsible for generating new mutations because the majority of our ‘positively selected sites’ demonstrate these properties. By definition, our previous analysis [1] identified selected sites if they had an increased non-synonymous to synonymous rate ratio. Perhaps then our ‘positively selected sites’ are simply an artefact of the DNA as we have described: regions of the DNA that experience increased rates of mutations based on a combination of sequence repeats and functional constraints at the protein level to produce non-synonymous substitutions. If that were true, then we would expect the proportion of mutations owing to SRaF to be the same between ‘neutral’ non-synonymous sites and the positively selected sites we identified above in the ND5 codons. These sites are considered neutral because under our PAML analysis, there were an equal (or nearly equal) number of synonymous and non-synonymous changes at these sites and/or the physico-chemical property was not significantly different among amino acid substitutions. Therefore, we used the same criteria to determine whether or not each neutral non-synonymous substitution from the same 14 taxa was a result of SRaF.

The mean proportion of SRaF at the 1723 neutral non-synonymous sites among our 237 taxa was 0.67 (table 2) with a standard deviation of 0.04. This suggests that the SRaF is an efficient means to generate neutral non-synonymous changes but that they do not explain all neutral non-synonymous changes because the proportion of positively selected sites that were probably SRaF induced was 0.97±0.04 (table 2). To determine whether SRaF was specific to non-synonymous sites, we calculated the proportion of SRaF-induced mutations at synonymous sites under our model and found that the mean value of those 5497 sites did not differ from neutral non-synonymous sites (table 2, *p*<0.910). By contrast, the proportion of positively selective sites that were SRaF-induced differed from neutral non-synonymous and synonymous sites that were induced by SRaF (table 2, *p*<0.0001).

**Table 2.** The number of sites with an SSR as part of the synonymous (syn-SSR) and non-synonymous (NS-SSR) codons that are owing to SRaF. (The proportion is also given for the non-synonymous (%SSR-NS), synonymous (%SSR-S) and positively selected (%SSR-Pos) sites that are owing to SRaF.)

species	NS-SSR	NS-no SSR	proportion SSR-NS	syn-SSR	syn-no SSR	proportion SSR-S	proportion SSR-Pos
*Anguilla*	44	24	0.65	258	134	0.66	1.0
Argentinoidei	165	121	0.58	383	231	0.62	1.0
Balaenopteridae	62	22	0.74	233	99	0.70	1.0
Caprinae	74	35	0.68	201	109	0.65	0.8
Delphinidae	55	34	0.62	206	99	0.68	1.0
*Grus*	59	23	0.72	197	94	0.68	1.0
Hominidae	182	83	0.69	336	151	0.69	1.0
*Hypsiglena*	65	28	0.70	274	88	0.76	1.0
Otariidae	75	36	0.68	263	93	0.74	1.0
*Oryzias latipes*	97	44	0.69	293	170	0.63	1.0
Phocidae	84	42	0.67	302	152	0.67	1.0
Salmondiae	38	19	0.67	228	108	0.68	1.0
*Takifugu*	22	14	0.61	169	120	0.58	1.0
Ursidae	124	52	0.70	340	166	0.67	1.0
mean			0.67			0.67	0.97
s.d.			0.04			0.04	0.04

## 5. Discussion

Our results indicate that recurrent positive Darwinian selection at functionally important sites in the ND5 protein of mitochondrial complex I in numerous taxa is a result of the properties inherent in the DNA sequence and constraint at the protein level. To return to our original analogy, the monkeys may be randomly hitting keys on the typewriters, but the typewriters are rigged to produce a greater number of mutations at specific regions of the DNA. By contrast, it appears that non-synonymous changes which are produced randomly (i.e. non-SRaF mutations) contribute little to the evolutionary process in these datasets because the sites that are produced without the SRaF model we describe occur only at neutral non-synonymous and synonymous sites. This is even more evident given that we probably overestimated the extent of non-SRaF mutation because we did not count sites as ‘SRaF-induced’ if they were adjacent to a sequence repeat; we included only those that were within or between two sequence repeats and it is likely that sites adjacent to a repeat are affected by the excision-repair process.

However, the sample size for the positively selected sites was considerably smaller than the sample size for the other two categories, so it is possible that simple random mutation from non-SRaF also produces variation on which positive selection can act. In addition, our neutral category of non-synonymous sites may include some sites that are under positive selection because one of the criterion that we used to identify these sites (the PAML analysis) is conservative and may not have detected some episodes of positive selection if they occurred in a single taxon or a few taxa within a group. Even taking these factors into account, random mutation is probably not the major source of variation on which Darwinian selection acts in this sample of mitochondrial genomes, but rather it acts on mutation produced by the processes described in our SRaF model.

Another important point to note is that because this process is occurring in mitochondrial DNA, it can act rapidly and occur at multiple levels. Eukaryotic animal cells contain hundreds to thousands of mitochondria, each with multiple copies of the mitochondrial genome. Selection could act among genomes within a mitochondrion, among mitochondria within a cell, among cells within tissues of an individual and among individuals within a population. In highly fecund species, selection could occur rapidly (although this would necessitate selection acting on germ-line cells).

It was previously reported that an individual primordial gamete in a female salmonid can hold billions of copies of mitochondrial DNA [22]. Therefore, selection for specific mitochondrial haplotypes in these species could occur within or among the gametes in an adult female salmon as she feeds at sea, and it could also occur within and among the fertilized embryos as they incubate in the gravel. This would suggest that selection could act rapidly. Indeed, it has been observed that mitochondrial genotypes can shift completely in a few generations in cows (*Bos taurus*) [23] and selection for specific haplotypes occurred in mass die-offs of the common murre (*Uria aalge*) in Alaska [24]. However, we note that the predominant force appears to be the maintenance of clonal haplotypes within a population 25,26.

In conclusion, it appears that the mutational processes which drive evolution are not random, but rather are the result of properties of the DNA itself in combination with functional constraint at the protein level. Our example here is limited to coding DNA in the mitochondrial genome, so it is not known whether this is also the process that drives molecular evolution of coding DNA in the nuclear genome and it is also possible that simple random mutation plays a larger role in the evolutionary process in other nuclear and mitochondrial genomic regions of DNA. A similar analysis of nuclear-encoded proteins would probably prove informative but the generation of the data here was labour-intensive and future efforts would be best done with an algorithm to identify sequence repeats. Current software exists to identify simple repeats but could not identify some of the more complex repeat units that we observed.

Additionally, much of the evolution process occurs at the gene-expression level among species and populations [27], so other models may apply in those regions of the genome. One could imagine a similar model to our SRaF in which sequence repeats still generate mutation owing to strand-slippage, but the constraint would lie in preserving DNA sequences that involve binding of transcription factors or regulation of mRNA molecules as a result of changes in the 5^′^- and 3^′^-untranslated regions rather than protein structural and functional constraint. If our model and evolutionary predictions are correct, then the extent of the SRaF system at the mitochondrial locus could be used to identify those species that will best respond to rapidly changing environments and prioritize those which need the most attention with regards to their potential for bioenergetic adaptation.

## Data accessibility

Data available from the Dryad Digital Repository: http://dx.doi.org/10.5061/dryad.m06t1.
